# Production of Recombinant* Trichoderma reesei* Cellobiohydrolase II in a New Expression System Based on* Wickerhamomyces anomalus*

**DOI:** 10.1155/2017/6980565

**Published:** 2017-08-30

**Authors:** Dennis J. Díaz-Rincón, Ivonne Duque, Erika Osorio, Alexander Rodríguez-López, Angela Espejo-Mojica, Claudia M. Parra-Giraldo, Raúl A. Poutou-Piñales, Carlos J. Alméciga-Díaz, Balkys Quevedo-Hidalgo

**Affiliations:** ^1^Institute for the Study of Inborn Errors of Metabolism, Facultad de Ciencias, Pontificia Universidad Javeriana, Bogotá, Colombia; ^2^Departamento de Química, Facultad de Ciencias, Pontificia Universidad Javeriana, Bogotá, Colombia; ^3^Unidad de Proteómica y Micosis Humanas, Grupo de Enfermedades Infecciosas, Departamento de Microbiología, Facultad de Ciencias, Pontificia Universidad Javeriana, Bogotá, Colombia; ^4^Grupo de Biotecnología Ambiental e Industrial (GBAI), Departamento de Microbiología, Facultad de Ciencias, Pontificia Universidad Javeriana, Bogotá, Colombia

## Abstract

Cellulase is a family of at least three groups of enzymes that participate in the sequential hydrolysis of cellulose. Recombinant expression of cellulases might allow reducing their production times and increasing the low proteins concentrations obtained with filamentous fungi. In this study, we describe the production of* Trichoderma reesei *cellobiohydrolase II (CBHII) in a native strain of* Wickerhamomyces anomalus*. Recombinant CBHII was expressed in* W. anomalus* 54-A reaching enzyme activity values of up to 14.5 U L^−1^. The enzyme extract showed optimum pH and temperature of 5.0–6.0 and 40°C, respectively. Enzyme kinetic parameters (*K*_*M*_ of 2.73 mM and* V*max of 23.1 *µ*M min^−1^) were between the ranges of values reported for other CBHII enzymes. Finally, the results showed that an enzymatic extract of* W. anomalus* 54-A carrying the recombinant* T. reesei* CBHII allows production of reducing sugars similar to that of a crude extract from cellulolytic fungi. These results show the first report on the use of* W. anomalus* as a host to produce recombinant proteins. In addition, recombinant* T. reesei* CBHII enzyme could potentially be used in the degradation of lignocellulosic residues to produce bioethanol, based on its pH and temperature activity profile.

## 1. Introduction

Cellulases are enzymes from the glycoside hydrolase family (EC 3.2.1.-) that are expressed by a broad spectrum of bacteria and fungi strains [1, 2]. Among these microorganisms, white- and brown-rot fungi are considered the major producers of cellulases, including* Trichoderma *sp.,* Aspergillus *sp.,* Schizophyllum commune*, and* Volvariella volvacea* [1, 2]. Cellulase is a family of at least three groups of enzymes: endo-(1,4)-*β*-D-glucanase (EC 3.2.1.4, EG), exo-(1,4)-*β*-D-glucanase (EC 3.2.1.91, CBH), and *β*-glucosidases (EC 3.2.1.21, BG) [3]. These enzymes act sequentially on the cellulose hydrolysis: (1) EG randomly attacks on O-glycosidic bonds resulting in glucan chains of different lengths, (2) CBH acts on glucan chains ends to release *β*-cellobiose, and (3) BG hydrolyzes the *β*-cellobiose to produce glucose [3].

*Trichoderma reesei* is one of the most important cellulases producing filamentous fungi, since it contains the core enzymes necessary for the complete hydrolysis of lignocellulose material [4]. Among these enzymes, cellobiohydrolases have been shown to be one of the most important components within this process [4]. Enzymes involved in hydrolysis of cellulose polymer molecules have been recombinantly expressed [5], which might allow reducing the production times and increasing the low proteins concentrations obtained with filamentous fungi. Recombinant expression might also facilitate the scaling up of the enzyme production, as well as the implementation of Simultaneous Saccharification and Fermentation (SSF) and Separate Hydrolysis and Fermentation (SHF) processes.* Saccharomyces cerevisiae* has been used for the production of recombinant CBH from* Phanerochaete chrysosporium* [6] and* Talaromyces emersonii* [7], while* T. reesei* CBHII has been expressed in* S. cerevisiae* [8, 9],* P. pastoris* [10, 11], and* Y. lipolytica* [11].

Conventional microbial systems (e.g.,* E. coli*,* S. cerevisiae*, and* P. pastoris*) have allowed the production of a long list of recombinant proteins [12]. However, there is a growing interest in the identification of new hosts that allow the production of high-quality or cost-efficient recombinant proteins [13]. Recently, a native strain of* Wickerhamomyces anomalus* was isolated from sugar cane bagasse at Puerto López, Meta, Colombia (unpublished results).* W. anomalus* is a yeast that has been isolated from different sources [14], and its reported applications include food biopreservation, bioremediation, and production of phytases and biofuels [14, 15]. However, to the best of our knowledge, there are no reports showing its use as host to produce recombinant proteins.

In this paper, we describe the production of recombinant cellobiohydrolase II (CBHII, EC 3.2.1.91) from* T. reesei* using the native strain* W. anomalus* 54-A. Furthermore, the effects of pH and temperature on enzyme activity were characterized, as well as the kinetic properties of the enzyme and its application on the hydrolysis of a lignocellulose material. Overall, the results showed the potential of* W. anomalus* 54-A as host to produce recombinant proteins and showed that recombinant* T. reesei* CBHII has similar characteristics to those reported for the wild-type enzyme.

## 2. Materials and Methods

### 2.1. Microorganism, Culture Media, and Vectors

*W. anomalus* 54-A was previously isolated from sugar cane bagasse at Puerto López, Meta, Colombia. Initially, the microorganism was identified by carbon assimilation profile using the API 20C AUX kit (bioMérieux, Marcy l'Etoile, France). Further microorganism identification was done by amplification of internal transcribed spacer (ITS), using the primers ITS1 5′-TCCGTAGGTGAACCTGCGG-3′ and ITS4 5′-TCCTCCGCTTATTGATATGC-3′ and* Pfu* DNA polymerase (Thermo Fisher Scientific Inc., San Jose, CA, US), under manufacturer instructions. Identity of* W. anomalus* 54-A was finally confirmed by MALDI-TOF analysis (Unidad de Investigación en Proteómica y Micosis Humana, Pontificia Universidad Javeriana).

Plasmid pKS2-ST (Dualsystems Biotech, Schlieren, Switzerland) was used as expression vector. In this vector, protein expression is regulated by the alcohol dehydrogenase II promoter (EC 1.1.1.1, ADH2), while the secretion signal of the* Saccharomyces cerevisiae* invertase SUC2 mediates the secretion of the recombinant protein.* Escherichia coli* DH5 was used for cloning purposes, which was cultured in Luria-Bertani (LB) medium supplemented with 100 mg mL^−1^ ampicillin. Yeast cultures were performed in YPD supplemented with 500 mg mL^−1^ geneticin for the selection of clones and 60 mg mL^−1^ for protein expression cultures.

### 2.2. Expression Vector and Recombinant Strains

Cellobiohydrolase II gene* (cbhII)* from* T. reesei *(GenBank Number GU724763.1) was previously codon-optimized for its expression in* S. cerevisiae *by GeneArt™ (Thermo Fisher Scientific Inc.) (unpublished data). The sequence encoding for the CBHII signal peptide was identified by using SignalP [16] and removed from the gene sequence. Comparison of the codon usage tables of* S. cerevisiae* and* W. anomalus* showed a similar profile between both microorganisms (Supplementary Figure  1 in Supplementary Material available online at https://doi.org/10.1155/2017/6980565) Two codons (CGC and CGG), which encode Arg (14 residues, 3%, in CBHII) showed significant differences between both microorganisms. Nevertheless, the other two Arg codons have a comparable usage between* S. cerevisiae* and* W. anomalus*. In this sense, this codon optimized sequence was used for the CBHII expression in* W. anomalus* 54-A. Codon optimized* cbhII* gene was inserted downstream of the SUC2 secretion signal in the vector pKS2-ST, to produce pKS2-ST::CBHII (Supplementary Figure  2). The pKS2-ST::CBHII vector was used to transform competent cells of* W. anomalus* 54-A by electroporation, using the Gene Pulser electroporation system Xcell™ (Bio-Rad Laboratories) at 1400 V and 200 Ω. The* W. anomalus* 54-A clones were selected in YPD medium supplemented with 500 mg mL^−1^ geneticin. Clones were confirmed by PCR using the primers 5′-GAGGAGAGCATAGAAATGGGG-3′ and 5′-CAGCAGTAGCCATAGCACCA-3′, which amplify a fragment from* cbhII* gene. The PCR-positive clones were evaluated at 55 mL scale, according to vector pKS2-ST manufacturer's instructions (Dualsystems Biotech). Briefly, each clone was incubated for 10 h in 10 mL YPD, after which 15 mL of fresh YPD medium was added. After 14 h incubation, 30 mL of fresh YPD medium was added to reach a final culture volume of 55 mL. After 6 h of incubation, it was expected that the glucose was exhausted, and this time was considered as the beginning of the induction phase. 1 mL aliquots were taken every 24 h for 96 h to measure extracellular enzyme activity and cell density. All the assays were performed in triplicate at 28°C and 180 rpm. Residual glucose quantitation was carried out by DNS method [17]. Crude extracellular fractions of the clone with the highest enzyme activity were loaded and processed by SDS-PAGE.

### 2.3. Evaluation of Carbon Sources

The clone that showed the highest enzyme activity was used to evaluate the effect of carbon source (i.e., glucose and glycerol) on the enzyme production. Cultures were carried out in 2% (w/v) tryptone and 1% (w/v) yeast extract, supplemented with glycerol and glucose in different concentrations: 2% (w/v) glucose, 1% (w/v) glucose, 1% (w/v) glycerol, 2% (w/v) glucose, 1% (w/v) glycerol, and 2% (w/v) glycerol. All cultures were supplemented with 60 mg mL^−1^ geneticin.

### 2.4. Culture at 2.4 L Scale

The* W. anomalus* 54-A clone that showed the highest CBHII activity at 55 mL scale was scaled up to 2.4 L in a 3.7 L Bioengineering KFL2000 bioreactor. Cultures were done in modified YPD medium [2% (w/v) tryptone, 1% (w/v) yeast extract, 1% (w/v) glucose, and 1% (w/v) glycerol] supplemented with 60 mg mL^−1^ geneticin. Briefly, 2 mL from the cell bank was inoculated into 18 mL of culture medium and incubated for 24 h at 28°C and 180 rpm. The preinoculum was used to inoculate 180 mL of fresh culture medium and incubated for 24 h at 28°C and 180 rpm. Finally, the inoculum was used to inoculate 1000 mL of fresh culture medium at the bioreactor. After 15 h culture, 1200 mL of fresh medium was added to reach a final volume of 2.4 L and cultured during additional 96 h at 28°C, 400 to 800 rpm, and pH 6.0.

### 2.5. Cellobiohydrolase Activity Assay

The CBHII activity was carried out as previously described [18, 19], using 5 mM p-nitrophenyl *β*-D-cellobioside (pNPC, Sigma-Aldrich) in sodium acetate buffer 50 mM, pH 5.0. One enzyme unit was defined as the amount of enzyme releasing 1 *μ*mol of p-nitrophenol per min, and the activity was expressed as U L^−1^.

### 2.6. Characterization of Recombinant CBHII

The CBHII enzyme extract was evaluated at different pH and temperature conditions, using pNPC. To evaluate the effect of pH and temperature on enzyme activity, the enzyme activity assay was performed at 3.0, 4.0, 5.0, 6.0, 7.0, and 8.0 ± 0.2 and at 30, 40, 50, 60, and 70°C for 1 h. The reactions were stopped and read as described above. Apparent kinetic parameters (*K*_*M*_ and* V*max) were estimated for the crude extract by using pNPC between 0 and 6.5 mM and fitting the experimental data to a Michaelis-Menten model using the software GraphPad PRISM 6.0.

### 2.7. *Chrysanthemum* Wastes Degradation Assay

The effect of recombinant CBHII on cellulose hydrolysis was evaluated by using* Chrysanthemum* wastes, as previously described [18, 19], with modifications.* Chrysanthemum* wastes were autoclaved at 121°C for 15 min. Experiments were performed in substrate submerged cultures carried out in 100 mL flasks in a rotatory shaker at 150 rpm and 30°C, for 20 days. Flasks contained 1%* Chrysanthemum* wastes in 40 mL of the following enzyme crude extracts: (1) cellulolytic and hemicellulolytic extract from a* Penicillium *sp. culture (hereafter Ce-Hem extract), (2) concentrated recombinant CBHII extract from* W. anomalus* 54-A (hereafter rCBHII), and (3) a 1 : 1 Ce-Hem : rCBHII extracts mixture. Control culture was carried out by using 40 mL of distilled water. All cultures were carried out with 10 mM sodium azide, 0.1% tween 80, and pH 5.0. The response variable was the concentration of reducing sugars as measured by DNS method [17]. Results are reported as g L^−1^ of reducing sugars after subtraction of the results obtained with the control cultures, and normalized by the initial unis of CBHII present in each extract. Production of Ce-Hem extract was carried out by using a* Penicillium *sp. strain (Collection of Microorganisms of Pontificia Universidad Javeriana). For this purpose, 20 mL of a 10^6^ conidia mL^−1^ suspension was inoculated in 200 mL of a rice straw medium without nitrogen source and incubated for 8 days at 28°C and 120 rpm. The crude extract was obtained by centrifugation and sequential filtrations through Whatman paper Number 42 and 0.45 and 0.22 *μ*m polyether sulphone membranes (Pall Corp, Port Washington, NY, USA). Concentrated recombinant CBHII extract from* W. anomalus* 54-A was produced by using a 2.4 L culture of the* W. anomalus* 54-A Celo-3.2 clone, as described above. Culture medium was centrifuged at 8000*g* and filtered sequentially through Whatman paper Number 42 and 0.45 and 0.22 *μ*m polyether sulphone membranes (Pall Corp). Permeate was ultrafiltered through a 30 kDa cut-off membrane (Millipore, Billerica, MA, USA), until reaching a final volume of 20 mL. CBHII activity in both extracts was assayed as described above. The *β*-glucosidase (BG) activity in both extracts was assayed using 5 mM 4-nitrophenyl *β*-D-glucuronide (Sigma-Aldrich) as previously described [18, 19]. One BG unit was defined as the amount of enzyme releasing 1 *μ*mol of p-nitrophenol per min, and the activity was expressed as U L^−1^. The endoglucanase (EG) activity in both extracts was assayed using 2% carboxymethylcellulose (Sigma-Aldrich) [18, 19] and the DNS method for quantitation of reducing sugars [17]. One EG unit was defined as the amount of enzyme releasing 1 *μ*mol of reducing sugars, equivalent to glucose, per min [20], and the activity was expressed as U L^−1^.

### 2.8. Statistical Analysis

Differences between groups were tested for statistical significance by using one-way ANOVA. An error level of 5%  (*p* < 0.05) was considered significant. All analyses were performed using the software GraphPad PRISM 6.0.

## 3. Results and Discussion

### 3.1. Production of Recombinant CBHII in* W. anomalus* 54-A

In this study, we evaluated the production of the recombinant CBHII in the native yeast strain 54-A, previously isolated from sugar cane bagasse from Puerto López, Meta, Colombia. This strain showed a carbon assimilation profile (API 20C AUX, bioMérieux) different from that observed for* S. cerevisiae* strains, and it was identified as* Pichia anomala* (recently renamed as* Wickerhamomyces anomalus* [21]) with a 94.5% ID and 0,25* T* value. Identity of this strain was further confirmed as* W. anomalus* through ITS amplification (GenBank accession number KX676490, Supplementary Figure  3) and MALDI-TOF analysis (Supplementary Figure  4).* W. anomalus* is a yeast that have been isolated from different sources, such as food- and feed-related systems, insects, and human clinical samples. This yeast has a wide robustness to environmental stresses conditions like extreme pH or low water activity [14]. Biotechnological applications of* W. anomalus* strains include biopreservation agent due to antimicrobial activity against variety of microorganisms in fruits and cereals and production of enzymes (e.g., phytases), biofuels, and bioremediation [14, 15]. However, to the best of our knowledge, there are no reports of the use of this yeast as a host to produce recombinant proteins.

**Figure 1:**
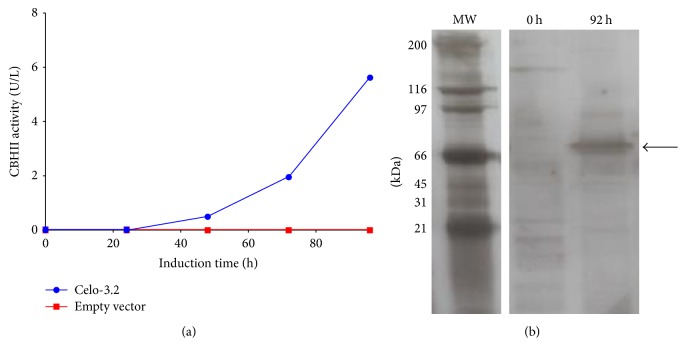
Recombinant* T. reesei* CBHII produced in* W. anomalus* 54-A. (a) PCR-positive clones of* W. anomalus* 54-A were evaluated at 55 mL scale and the activity of recombinant* T. reesei* CBHII was assayed in the extracellular fraction. Among the four PCR-positive clones, only* W. anomalus* 54-A Celo 3.2 clone showed CBHII activity. No activity was observed in* W. anomalus* 54-A transfected with the empty vector. Each clone was evaluated in triplicate. (b) Crude extracellular extracts from* W. anomalus* 54-A Celo 3.2 at 0 and 92 h were evaluated by SDS-PAGE, followed by silver staining. The arrow indicates the recombinant CBHII.

Four clones were obtained after transformation of* W. anomalus* 54-A with the vector pKS2-ST::CBHII (Celo-2.2, Celo-3.2, Celo-4.2, and Celo-5.1), which were confirmed by PCR. The* ADH2* gene promoter is repressed in the presence of glucose, since this metabolite inhibits the expression of the alcohol dehydrogenase regulator protein (Adr1) that is an activator of the* ADH2* gene. On the other hand, under glucose depletion conditions, the expression of Adr1 is increased leading to the induction of the* ADH2* gene [22]. In this sense, we first determined the residual glucose during the culture of* W. anomalus* 54-A pKS2-ST::CBHII clones, to establish the beginning of the induction phase. We observed, for all the evaluated clones, that, at the final culture step, the glucose was consumed within the first 6 h of incubation (Supplementary Figure  5). In this sense, this point was considered as the beginning of the induction phase, which agrees with previous reports of the production of recombinant proteins in yeast under the control of the* ADH2* gene promoter [22, 23]. Under these culture conditions, extracellular CBHII activity was only detected in* W. anomalus* 54-A Celo-3.2 clone, reaching a final enzyme activity of 5.7 U L^−1^ after 96 h of induction (Figure 1(a)). As expected, CBHII activity was not detected in* W. anomalus* 54-A transfected with the empty vector.

As was mentioned above,* T. reesei* CBHII has been expressed in* S. cerevisiae* [8, 9],* P. pastoris* [10, 11], and* Y. lipolytica* [11]. Recombinant CBHII produced in* S. cerevisiae* showed an activity of 25 S.I. U mL^−1^ using barely *β*-glucan as substrate [8], while recombinant CBHII produced in* P. pastoris* GS115 showed an enzyme activity of 5.84 U mL^−1^ at 96 h using microcrystalline cellulose PH101 as substrate [10]. In* P. pastoris* X-33 and* Y. lipolytica* POld, extracellular recombinant CBHII activities were 0.25 and 0.36 U mL^−1^, respectively, using phosphoric acid swollen cellulose (PASC) prepared from Avicel PH-101 as substrate [11]. The differences in promoters, secretion signals, hosts, and enzyme activity substrates limit the comparison between these results and those obtained for recombinant CBHII produced in* W. anomalus* 54-A. However, a crude culture broth from* Penicillium *sp. containing wild-type cellulase and hemicellulase enzymes (see* Chrysanthemum* Wastes Degradation Assay) showed a CBHII activity of 8.5 U L^−1^ using the pNPC substrate, which was similar to that observed for the recombinant CBHII produced in* W. anomalus* 54-A. Nevertheless, these results are lower than those observed for erlenmeyer flask cultures of* Pleurotus ostreatus*, which showed an activity of 445.3 ± 27.6 U L^−1^ using the pNPC substrate [18, 19].

SDS-PAGE analysis of extracellular fraction of* W. anomalus* 54-A Celo-3.2 showed that recombinant CBHII has an apparent molecular weight of about 66 kDa (Figure 1(b)). This recombinant CBHII is higher than the wild-type enzyme produced by* T. reesei *(50–58 kDa) [24]. However, recombinant CBHII produced in* W. anomalus 54-A* has a molecular weight similar to that reported for a CBHII produced in* P. pastoris *X33 and* Y. lipolytica *POld (~63–65 kDa) [11] but higher than that reported for the enzyme produced in* P. pastoris *GS115 (~58 kDa) [10]. Differences in the molecular weight between wild-type and the recombinant counterpart could be associated with the hypermannosylation observed in yeast, while the differences in the molecular weight among the recombinant CBHII could be associated with several factors of the culture conditions that affect the expression of glycosyltransferases [25].

### 3.2. Evaluation of Carbon Sources

To improve the production of recombinant CBHII, two glucose and glycerol concentrations were evaluated. First, 1% w/v glucose was evaluated, which is lower than the concentration suggested by the vector manufacturer (2% w/v). We hypothesize that 1% w/v glucose would be depleted faster than 2% w/v, allowing a reduction in the induction time and a longer and higher enzyme production [22]. In addition, glycerol, alone or in combination with glucose, was also evaluated as a carbon source, since it was expected that glycerol would be used for yeast growth after depletion of glucose, increasing the production of the recombinant enzyme.

**Figure 2:**
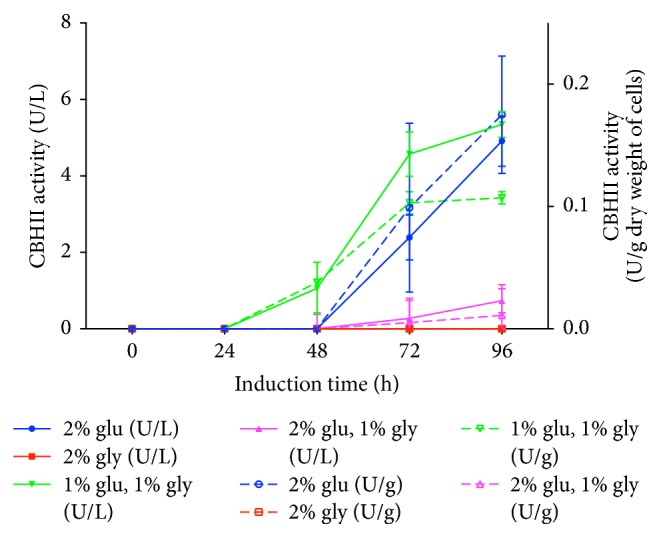
Effect of the carbon source on the production of recombinant* T. reesei* CBHII. Production of recombinant CBHII was carried out at 55 mL with 2% (w/v) glucose (circle), 2% (w/v) glycerol (square), 2% (w/v) glucose, 1% (w/v) glycerol (up triangle), and 1% (w/v) glucose, 1% (w/v) glycerol (down triangle). Enzyme activity is reported as U L^−1^ (continuous line) and U g^−1^ dry weight of cells (dashed line). Each experiment was done in triplicate.

Figure 2 shows the results of volumetric activity (U L^−1^) and enzyme activity normalized by biomass (U g^−1^ dry weight of cells). A similar activity profile was observed between volumetric and normalized enzyme activities, suggesting that the production of recombinant CBHII was associated with yeast growth. In fact, the profile of recombinant CBHII production was similar to that previously reported for the ADH2 promoter, with a complete induction in the late stationary phase (>36 h culture) [22]. The results showed that in 2% (w/v) glycerol cultures no CBHII activity was detected, suggesting that the glycerol was used for cell growth or that the ADH2 promoter needs to sense the glucose depletion to induce the gene expression. On the other hand, lower CBHII activity was observed at 2% (w/v) glucose, 1% (w/v) glycerol, cultures compared to that obtained with 2% (w/v) glucose (*p* < 0.001). Finally, the highest CBHII activity was observed at 1% (w/v) glucose, 1% (w/v) glycerol, although this was not statistically different regarding the levels obtained with 2% (w/v) glucose (*p *> 0.05). However, 1% (w/v) glucose, 1% (w/v) glycerol, allowed an earlier detection of the enzyme activity than that observed with 2% (w/v) glucose. In this sense, 1% (w/v) glucose, 1% (w/v) glycerol, was selected to produce recombinant CBHII at 2.4 L scale. The reasons of the effect of glycerol on CBHII activity are unknown, and further investigations should be carried out to clarify this aspect. Nevertheless, it is important to mention that there is a wide variation among yeasts, and even strains, regarding glycerol metabolism (i.e., uptake and catabolism) [26]. Although the metabolic pathways and the uptake and regulation (e.g., catabolic repression) process have been widely described for* S. cerevisiae*, limited information is available for other yeasts [26]. In this sense, the effect of glycerol on the changes of enzyme activity levels might be associated with the specific glycerol metabolism of* W. anomalus* 54-A.

### 3.3. Production of Recombinant CBHII at 2.4 L Scale

**Figure 3:**
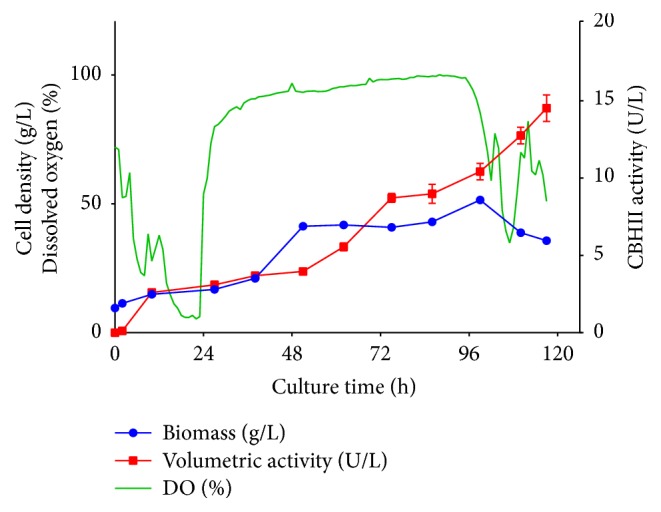
Production of recombinant* T. reesei* CBHII in* W. anomalus* 54-A at 2.4 L scale. Recombinant* T. reesei* CBHII was produced at 2.4 L with 1% (w/v) glucose and 1% (w/v) glycerol. Production was followed by 117 h after the final feeding step. Dissolved oxygen (%), cell density (gL^−1^), and extracellular enzyme activity (U L^−1^) are reported.

The production of recombinant CBHII enzyme at 2.4 L scale was carried out for 117 h after the final feeding step (see Materials and Methods).  Figure 3 shows the behavior of dissolved oxygen (DO) during the production of recombinant CBHII at bioreactor scale, which could be associated with the consumption of the carbon sources. The CBHII activity was detected from the first 2 h of culture (0.12 ± 0.08 U L^−1^). This activity significantly increased after 10 h (2.6 ± 0.09 U L^−1^) and continuously kept increasing to reach a final enzyme activity of 14.5 ± 0.86 U L^−1^, which was 2.7-fold higher than that observed at 55 mL scale. These results might suggest that, under controlled culture conditions, the strict repression of the ADH2 promoter might be affected allowing the expression of the recombinant protein even in the presence of residual glucose.

### 3.4. Characterization of CBH Recombinant Extract

**Figure 4:**
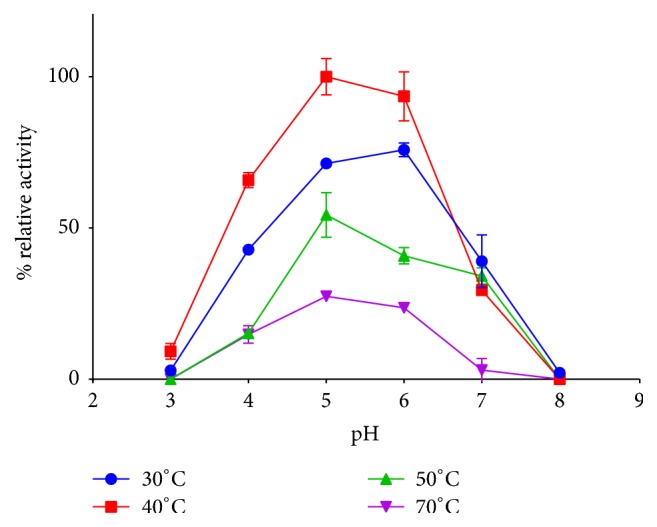
Effect of temperature and pH on reaction of recombinant* T. reesei* CBHII. The enzyme activity of recombinant* T. reesei* CBHII produced in* W. anomalus* 54-A was evaluated at different pH and temperatures reactions condition. Each experiment was carried out in triplicate. Activity is reported as % Relative Activity as compared with the highest activity obtained in the experiment (40°C, pH 5).

The effect of temperature and pH on the enzyme activity of the recombinant CBHII produced in* W. anomalus* 54-A was evaluated. In this work, crude extract rather than the purified recombinant CBHII was used for characterization, since this crude extract would be used in the production of bioethanol by the degradation of* Chrysanthemum* residues [19]. As observed in Figure 4, optimum reaction temperature for recombinant CBHII crude extract was 40°C (*p* < 0.001), while the highest enzyme activities were observed at pH 5.0 and 6.0 ± 0.2 at all evaluated temperatures (*p* < 0.001). On the other hand, at pH 3.0 and 8.0 ± 0.2, a marked decrease in the enzymatic activity was observed regardless of the reaction temperature.

Using microcrystalline cellulose PH101 as substrate, recombinant CBHII produced in* P. pastoris* showed an optimum reaction pH and temperature of 5.0 ± 0.2 and 50°C, respectively. In addition, this recombinant enzyme showed a significant loss of activity at pH of 3.0, 7.0, and 8.0 and at 30°C and temperatures above 65°C [10]. Recombinant CBHII enzyme produced in* P. pastoris* and* Y. lipolytica* showed an optimum pH between 5.0 and 6.0 and at a temperature of 60°C. Recombinant CBHII produced in* P. pastoris* and* Y. lipolytica *also showed rapid inactivation at pH and temperatures above 7.0 and 60°C, respectively, while at temperatures below 40°C, an 80% loss in the enzyme activity was observed [11]. In this sense, recombinant* T. reesei *CBHII produced in* W. anomalus* 54-A has a reaction pH profile similar to that reported for recombinant CBHII enzymes expressed in other yeast. Nevertheless, it seems that the optimum temperature of reaction differs among the recombinant CBHII enzymes, which might be associated with the substrate used for the enzyme activity assay or the posttranslational modifications carried out for each host. It is noteworthy that recombinant CBHII produced in* W. anomalus* 54-A only showed 30% reduction in enzyme activity at 30°C. Functionality at 30°C might allow the use of this enzyme in SSF processes for the production of bioethanol, since this temperature is also the optimum growth temperature for* S. cerevisiae *that is the preferable microorganism used during the fermentation stage of bioethanol production [27].

Kinetic parameters for the crude extract were estimated using pNPC as substrate (Supplementary Figure  6). Recombinant* T. reesei *CBHII produced in* W. anomalus* 54-A showed *K*_*M*_ and* V*max of 2.73 mM (est. error 0.26) and 23.1 *µ*M min^−1^ (est. error 0.89), respectively. This *K*_*M*_ value agrees with those reported for wild-type CBHII enzymes (EC 3.2.1.91) isolated from different microorganisms, which vary between 0.1 and 3.1 mM, depending on the source of the enzyme [28]. In the case of* T. reesei* CBHII enzymes there are no reports of kinetic parameters using pNPC substrate. Nevertheless, a wide variation in *K*_*M*_ values is observed (i.e., between 0.041 and 380 mM), depending on the substrate used for this estimation [28].

### 3.5. *Chrysanthemum* Wastes Degradation Assay

**Figure 5:**
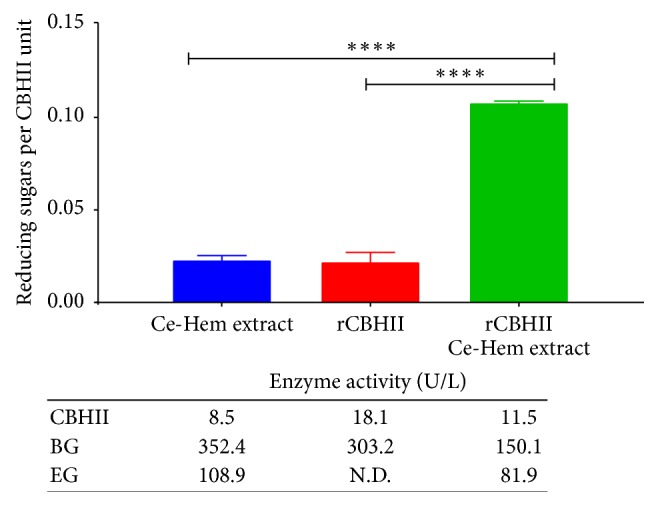
Chrysanthemum wastes degradation assay. The effect of recombinant CBHII on cellulose hydrolysis was evaluated by using* Chrysanthemum* wastes. Flasks that contained 1%* Chrysanthemum* wastes were incubated for 20 days at 150 rpm and 30°C with a crude culture broth from* Penicillium *sp. containing wild-type cellulase and hemicellulase enzymes (Ce-Hem extract), concentrated recombinant CBHII extract from* W. anomalus* 54-A (rCBHII), and a 1 : 1 Ce-Hem : rCBHII extracts mixture. Results are reported as g L^−1^ of reducing sugars per unit of CBHII after subtraction of the results obtained with the control cultures. *∗∗∗∗* = *p* < 0.0001. The table shows the enzyme activities of CBHII, *β*-glucosidase (BG), and endoglucanase (EG) present in the enzymatic extracts at the beginning of the assay.

To evaluate the effect of recombinant CBHII on cellulose hydrolysis,* Chrysanthemum *wastes degradation assay was performed using three enzyme extracts: (1) a crude culture broth from* Penicillium *sp. containing wild-type cellulase and hemicellulase enzymes (Ce-Hem extract), (2) a concentrated recombinant CBHII extract from* W. anomalus* 54-A (rCBHII extract), and (3) a 1 : 1 Ce-Hem : rCBHII extracts mixture. After treatment, 0.2 ± 0.02, 0.4 ± 0.01, and 1.25 ± 0.1 g L^−1^ of reducing sugars were observed for Ce-Hem, rCBHII, and 1 : 1 Ce-Hem : rCBHII extracts, respectively. However, when reducing sugar concentration was normalized by the CBHII units present in each extract at the beginning of the experiment (Figure 5), it was observed that rCBHII extract had production of reducing sugars similar to that obtained with the Ce-Hem extract (*p* > 0.05). Noteworthy, the use of a 1 : 1 Ce-Hem : rCBHII extracts mixture allowed an increase of about 5-fold, in the reducing sugars per CBHII unit, in comparison with the results obtained by using the Ce-Hem or concentrated recombinant CBHII extracts alone, suggesting a synergistic effect of the combination of these two enzymatic extracts. To understand the reasons of this synergistic effect, we measured the activity of *β*-glucosidase (BG) and endoglucanase (EG) in the extracts (Figure 5). Whereas BG was detected in the three evaluated extracts, EG was only detected in Ce-Hem and Ce-Hem : rCBHII extracts. Nevertheless, there was no correlation between BG and EG activity and the increase in reducing sugars observed with the Ce-Hem : rCBHII extract. In this sense, we hypothesize that the synergistic effect observed in the Ce-Hem : rCBHII treatment could be associated with the presence of other enzymes, such as lignin peroxidase, xylanase, laccase, and manganese peroxidase [29].

*Chrysanthemum *wastes degradation using enzymatic extracts with native or recombinant cellulases has not been previously reported. For this lignocellulosic waste, degradation with* P. ostreatus* showed a production of 9.6 g L^−1^ of reducing sugars [18]. The higher production of reducing sugars using* P. ostreatus* could be associated with the production of several lignocellulolytic enzymes by* P. ostreatus* such as laccases (up to 6 isoforms), manganese peroxidases, cellulases, and xylanases [30–32]. The presence of all these lignocellulolytic enzymes has shown an improvement in the delignification and reducing sugars production processes [29, 33]. In addition, the use of enzymatic extracts with recombinant cellulases, to degrade lignocellulosic materials, has been preceded by chemical or physical-chemical delignification treatments, such as alkaline [10] or steam [34] pretreatments. These pretreatments allowed higher reducing sugar levels than those reported in the present study (between 1.2 and 2.0 g L^−1^), showing the importance of lignin degradation to increase biomass digestibility [29]. Nevertheless, these results showed that an enzymatic extract of* W. anomalus* 54-A carrying a recombinant* T. reesei* CBHII allows production of reducing sugars similar to that of a crude extract from cellulolytic fungi, showing the potential of* W. anomalus* 54-A as a host to produce recombinant cellulases.

In conclusion, we reported the production of the recombinant* T. reesei* CBHII in* W. anomalus* 54-A strains. The results showed production of recombinant CBHII in* W. anomalus* 54-A, with enzyme activity levels of up to 14.5 U L^−1^. Recombinant CBHII showed optimum pH and temperature reaction of 5.0–6.0 ± 0.2 and 40°C, respectively, which were similar to those reported for other recombinant* T. reesei* CBHII enzymes.* Km* of this recombinant CBHII was between the ranges of values reported for other CBHII enzymes. The results showed that an enzymatic extract of* W. anomalus* 54-A carrying the recombinant* T. reesei* CBHII allows production of reducing sugars similar to that of a crude extract from cellulolytic fungi, showing the potential of* W. anomalus* 54-A as a host to produce recombinant cellulases. Noteworthily, this is the first report on the use of* W. anomalus *as a host to produce heterologous proteins. Further studies should be focused on the consolidation of* W. anomalus* as a platform to produce recombinant proteins such as the design of specific expression vectors and the generation of auxotrophic strains.

## Supplementary Material

Supplementary materials show: (1) Comparison of S. cerevisiae and W. anomalus codon usage, (2) Expression vector pKS2-ST::CBHII, (3) Wickerhamomyces anomalus 54-A identification by ITS amplification and MALDI-TOF, (4) Residual glucose during the culture of W. anomalus 54-A pKS2-ST::CBHII clones, and (5) Estimation of kinetic parameters for the recombinant T. reesei CBHII.

## Additional Points

*One-Sentence Summary.* This work represents the first report on the use of the yeast* Wickerhamomyces anomalus* as a host to produce heterologous proteins.
