# Supplementary material for: Production of Recombinant Trichoderma reesei Cellobiohydrolase II in a New Expression System Based on Wickerhamomyces anomalus
Source: Enzyme Res. 2017 Aug 30;2017:6980565. doi: 10.1155/2017/6980565 (PMC5603120; doi:10.1155/2017/6980565)
Supplement: Supplementary file 1 — Supplementary materials show: (1) Comparison of S. cerevisiae and W. anomalus codon usage, (2) Expression vector pKS2-ST::CBHII, (3) Wickerhamomyces anomalus 54-A identification by ITS amplification and MALDI-TOF, (4) Residual glucose during the culture of W. anomalus 54-A pKS2-ST::CBHII clones, and (5) Estimation of kinetic parameters for the recombinant T. reesei CBHII. [file 6980565.f1.pdf]

Supplementary Material

Production of recombinant *Trichoderma reesei* cellobiohydrolase II in a new expression system based on *Wickerhamomyces anomalus*

Dennis J. Díaz-Rincón[1], Ivonne Duque[1], Erika Osorio[1], Alexander Rodríguez-López[1,2], Angela Espejo-Mojica[1], Claudia M. Parra-Giraldo[3], Raúl A. Poutou-Piñales[4], Carlos J. Alméciga-Díaz[1,*], Balkys Quevedo-Hidalgo[4,*]

[1] Institute for the Study of Inborn Errors of Metabolism, Facultad de Ciencias, Pontificia Universidad Javeriana.

[2] Departamento de Química, Facultad de Ciencias, Pontificia Universidad Javeriana.

[3] Unidad de Proteómica y Micosis Humanas, Grupo de Enfermedades Infecciosas Departamento de Microbiología, Facultad de Ciencias, Pontificia Universidad Javeriana

[4] Grupo de Biotecnología Ambiental e Industrial (GBAI), Departamento de Microbiología, Facultad de Ciencias, Pontificia Universidad Javeriana.

*** Corresponding authors:**

Carlos Javier Alméciga-Díaz, Proteins Expression and Purification Laboratory, Institute for the Study of Inborn Errors of Metabolism, School of Sciences, Pontificia Universidad Javeriana, Kra 7 No. 43-82 Building 53, room 303A. Bogotá, Colombia. Tel: +57 1 320 8320 Ext 4140; Fax: +57 1 320 8320 Ext 4099; E-mail: cjalmeciga@javeriana.edu.co

Balkys Quevedo- Hidalgo, Laboratorio de Biotecnología Aplicada, Grupo de Biotecnología Ambiental e Industrial (GBAI), Departamento de Microbiología, Facultad de Ciencias, Pontificia Universidad Javeriana, Kra 7 No. 43-82 Building 50, room 120. Bogotá, Colombia. Tel: +57 1 320 8320 Ext 4030 E-mail: bquevedo@javeriana.edu.co

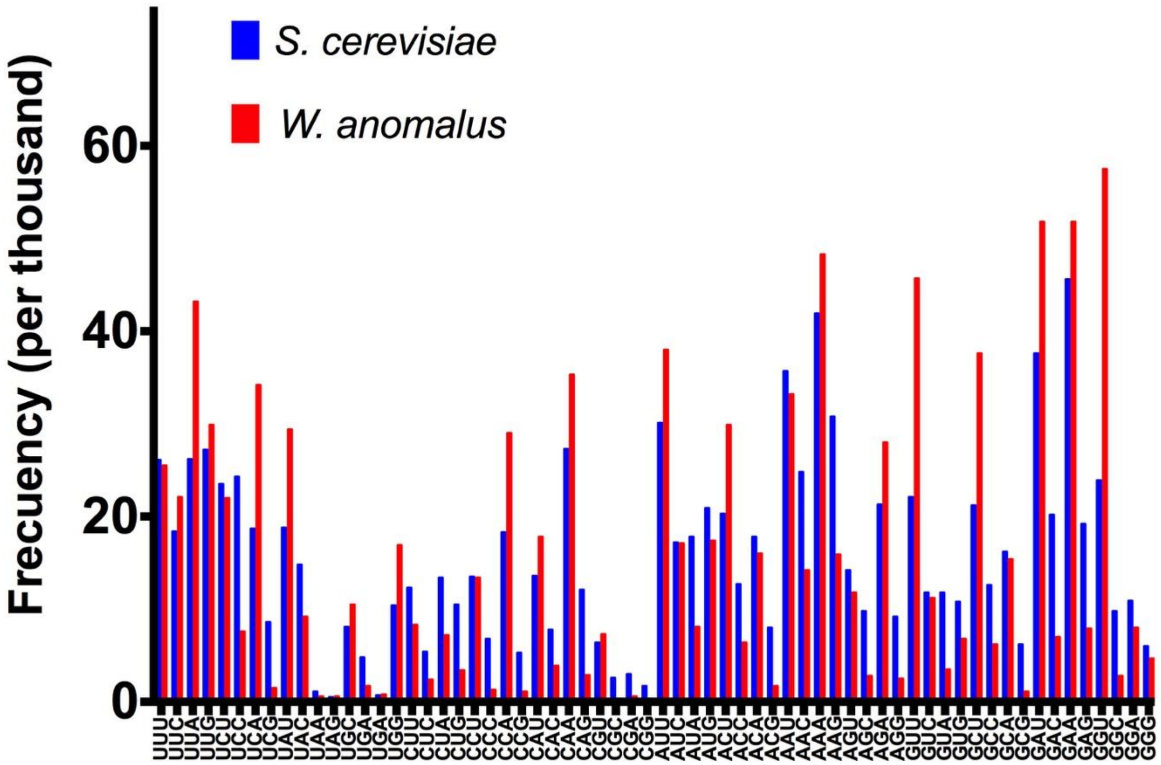

Supplementary Figure 1. Comparison of *S. cerevisiae* and *W. anomalus* codon usage. Codon usage tables for *S. cerevisiae* (14411 CDS's, 6534504 codons) and *W. anomalus* (26 CDS's, 12724 codons) were retrieved from the Codon Usage Database (http://www.kazusa.or.jp/codon/).

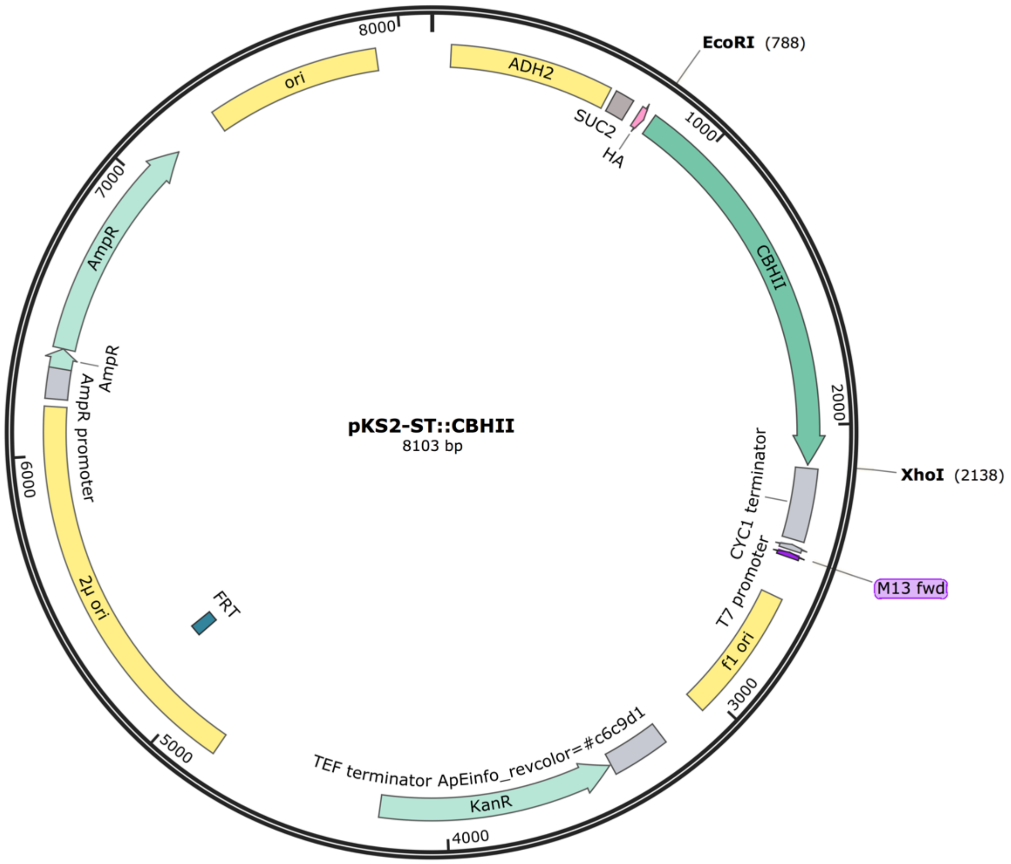

Supplementary Figure 2. Expression vector pKS2-ST::CBHII. Codon optimized *cbhII* gene was inserted downstream of the SUC2 secretion signal in the vector pKS2-ST, to produce pKS2-ST::CBHII**.** In this vector, protein expression is regulated by the alcohol dehydrogenase II promoter (E.C. 1.1.1.1, ADH2); while the secretion signal of the *Saccharomyces cerevisiae* invertase SUC2 mediates the secretion of the recombinant protein.

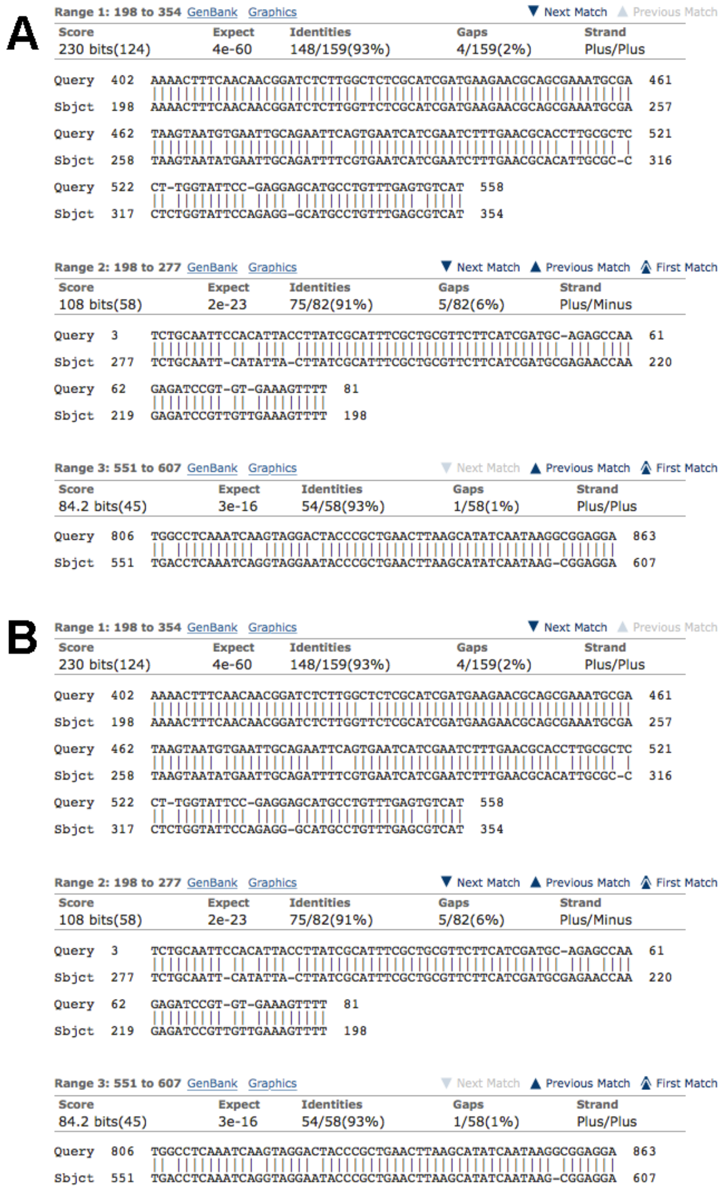

Supplementary Figure 3. ***Wickerhamomyces anomalus* 54-A identification by ITS amplification.** Identity of the strain 54-A was confirmed as *W. anomalus* through ITS amplification using the primers using the primers ITS1 5'-tccgtaggtgaacctgcgg-3' and ITS4 5'-tcctccgcttattgatatgc-3'. PCR product (GenBank accession No. KX676490) was sequenced and compared against reported ITR sequences for *W. anomalus* strains on Genbank using Blastn tool (http://blast.ncbi.nlm.nih.gov/Blast.cgi). Query: *W. anomalus* 54-A ITS; Sbjct: A) *Wickerhamomyces anomalus* strain MTCC 4133 small subunit ribosomal RNA gene, partial sequence; internal transcribed spacer 1, 5.8S ribosomal RNA gene, and internal transcribed spacer 2, complete sequence; and large subunit ribosomal RNA gene, partial sequence (Genbank No. HQ905435.1, E value: 4×10^{-60}), B) *Pichia anomala* strain CBS 605T 5.8S ribosomal RNA gene, partial sequence; internal transcribed spacer 2, complete sequence; 28S ribosomal RNA gene, partial sequence (Genbank No. AF218991.1, E value: 4×10^{-39}).

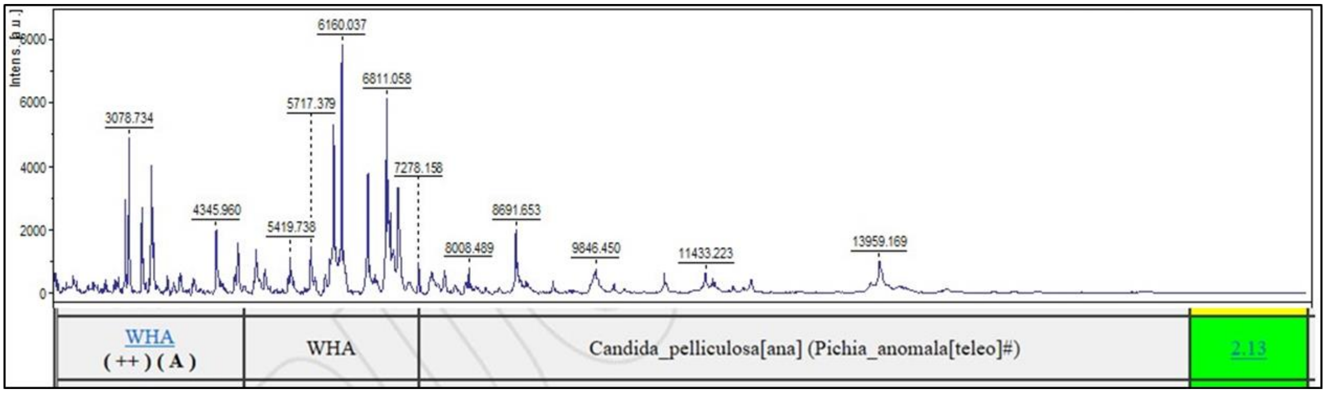

Supplementary Figure 4. ***Wickerhamomyces anomalus* 54-A identification with BIOTYPER BRUKER library for MALDI-TOF analysis of global cytoplasmic proteins.** Mass spectra was generated with the Microflex LT mass spectrometer (Bruker Daltonik GmbH) using the manufacturer's protocol. For this purpose, the yeast was cultured in YPD agar [2% (w/v) glucose, 1% (w/v) yeast extract, and 2% (w/v) tryptone], and colonies were suspended in molecular grade deionized water. Cell pellet was washed with ethanol, dried at 25 °C, and reconstituted in equal volumes of 70% (v/v) formic acid (Sigma-Aldrich, St. Louis, MO, USA) and acetonitrile (Sigma-Aldrich). One microliter of supernatant was spotted onto a 96-spot steel plate (Bruker Daltonik Ltd., East Milton, ON, Canada) and allowed to dry at 25 °C before addition of 1 mL of MALDI matrix (a saturated solution of a-cyano-4-hydroxycinnamic acid). The identification of the microorganism is based on the analysis of the global profile of mass spectrometry obtained from cytoplasmic proteins, mainly ribosomal and chaperon proteins. This profile is analyzed by comparison with the mass spectrometry for reference strains from the BIOTYPER BRUKER library. MALDI-TOF/MS results were then compared and scored according to the manufacturer's technical specifications as follows: correct genus and species identification (≥2.0), secure genus identification (1.7–2.0), and no reliable identification (<1.7).

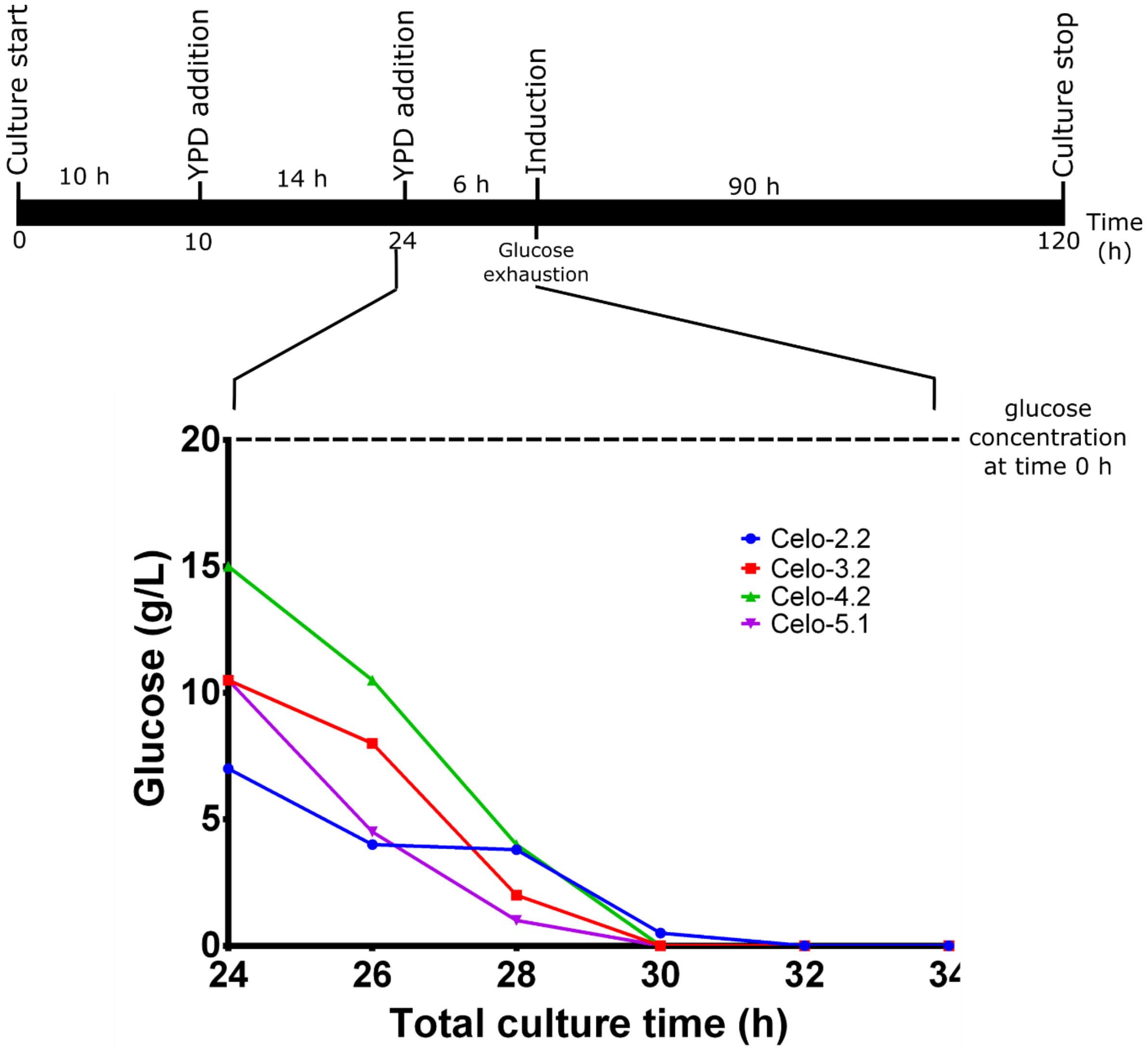

Supplementary Figure 5. Residual glucose during the culture of *W. anomalus* 54-A pKS2-ST::CBHII clones. *W. anomalus* 54-A was transformed with pKS2-ST::CBHII plasmid, and PCR-positive clones were cultured at 55 mL scale. Residual glucose quantitation was carried out by using DNS method after the last addition of fresh YPD medium. As mentioned in Materials and Methods, cultures were carried out according to plasmid manufacturer´s instructions (Dualsystems Biotech). This protocol includes: 1) a 10 ml inoculum in YPD cultured during 10 h; 2) the addition of 15 mL of fresh YPD after 10 h culture, and 3) after 14 h incubation, the addition of 30 mL of fresh YPD (upper figure). Since glucose consumption will depend on the growth of each clone, after the two additions of fresh YPD there will be differences in the residual glucose at the final step of the culture (show in the bottom figure). Dashed line at the bottom figure represents the glucose concentration at culture start.

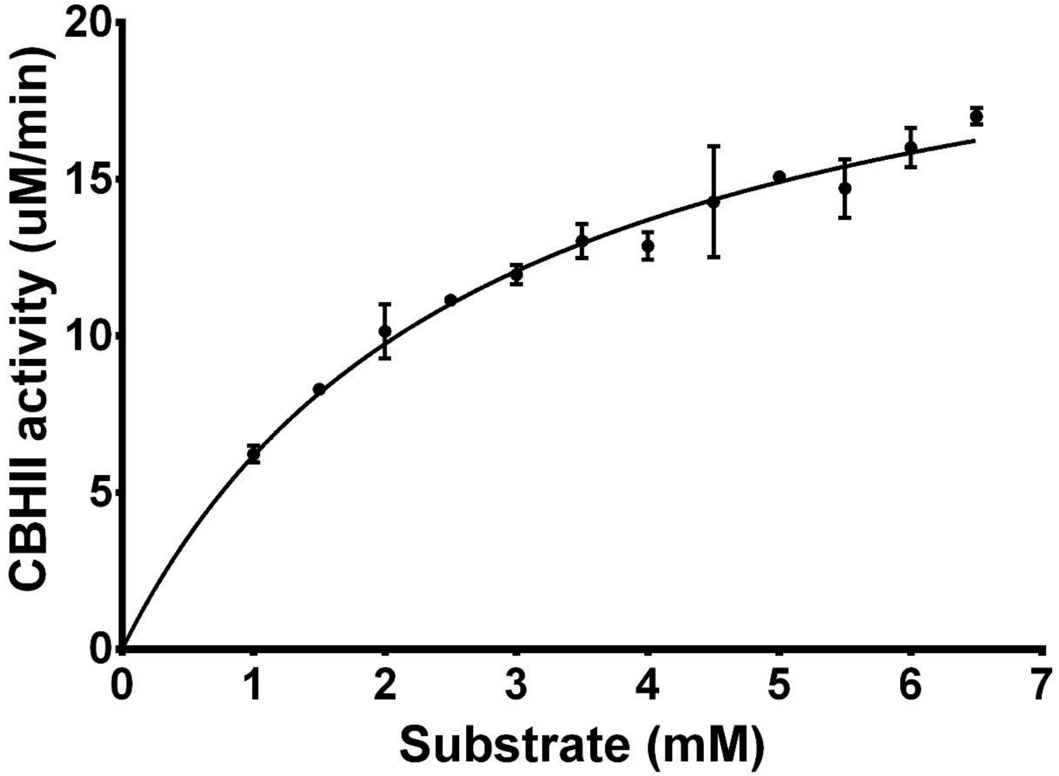

Supplementary Figure 6. Estimation of kinetic parameters for the crude extract of recombinant *T. reesei* CBHII produced in in *W. anomalus* 54-A. Apparent kinetic K_M and Vmax were estimated for the crude extract by using the pNPC substrate between 0 and 6.5 mM. K_M and V_{max} were calculated by fitting the experimental data to a Michaelis-Menten model using the software GraphPad PRISM 6.0. Each experiment was carried out by triplicate
